# Prolonged Use of Carnitine-Orotate Complex (Godex^®^) Is Associated with Improved Mortality: A Nationwide Cohort Study

**DOI:** 10.3390/jpm12121970

**Published:** 2022-11-28

**Authors:** Kye-Yeung Park, Sangmo Hong, Kyung-Soo Kim, Kyungdo Han, Cheol-Young Park

**Affiliations:** 1 Department of Family Medicine, Hanyang University College of Medicine, Seoul 04763, Republic of Korea; 2 Department of Internal Medicine, Hanyang University Guri Hospital, Hanyang University College of Medicine, Guri 11923, Republic of Korea; 3 Department of Internal Medicine, CHA Bundang Medical Center, CHA University School of Medicine, Seongnam 13497, Republic of Korea; 4 Department of Statistics and Actuarial Science, Soongsil University, Seoul 06978, Republic of Korea; 5 Department of Internal Medicine, Kangbuk Samsung Hospital, Sungkyunkwan University School of Medicine, Seoul 03181, Republic of Korea

**Keywords:** carnitine, cardiometabolic risk factors, epidemiologic study, metabolic diseases, mortality

## Abstract

Despite its hepatoprotective effects and favorable metabolic effects, the association between carnitine-orotate complex (Godex^®^) intake and mortality has never been investigated. We enrolled 13,413 adults who underwent national health examination and were prescribed the carnitine-orotate complex. Subjects were classified into three groups based on duration of using carnitine-orotate complex: <30, 30–180, and ≥180 days and were followed-up until 2019. Hazard ratios (HRs) and 95% confidence intervals (CIs) for all-cause mortality were estimated using Cox proportional hazards regression. During the follow-up period, 708 deaths were documented. Adjusted HR of mortality was 0.69 (95% CI 0.51–0.92) in those who used carnitine-orotate complex for ≥180 days compared to those who used it for <30 days. Use of carnitine-orotate complex for ≥180 days was associated with significantly reduced mortality in individuals with metabolic risk factors such as obesity, metabolic syndrome, dyslipidemia, and fatty liver than the shorter period of use. A significant interaction was observed in individuals with type 2 diabetes (HR 0.43, 95% CI 0.29–0.63, *p*-value 0.001). In this nationwide study, longer use of carnitine-orotate complex was associated with improved mortality compared to a shorter period of use, and the risk reductions were prominent in individuals with metabolic risk factors.

## 1. Introduction

Carnitine-orotate complex (Godex^®^) is an oral supplement that has shown efficacy in individuals with increased levels of liver enzyme and various liver diseases [1,2,3]. Recently, in vitro and in vivo studies have shown that carnitine-orotate complex can improve metabolic health such that improved glucose and lipid metabolism and significantly alleviated hepatic steatosis [4,5,6,7]. Furthermore, a recent experimental study elucidated the molecular mechanism by which carnitine-orotate complex ameliorates insulin resistance [6].

L-carnitine, the main component of carnitine-orotate complex, has previously been reported to improve mortality in patients with sepsis, chronic kidney disease, and heart failure [8,9,10,11]. A Japanese study reported that L-carnitine supplementation improved mortality in patients with liver cirrhosis (LC) [11]. However, no prior study has investigated the effects of using carnitine-orotate complex on mortality. Considering the beneficial metabolic effects of carnitine-orotate complex and their widespread use in general population, this prompted us to examine the association between the use of carnitine-orotate complex and the risk of mortality.

We hypothesized that use of carnitine-orotate complex would decrease the overall risk of mortality and that this effect might be associated with duration of use, as the prescribed dose of carnitine-orotate complex is relatively fixed. In addition, we hypothesized that the effects of carnitine-orotate complex on mortality would be different in individuals with metabolic risk factors, given its metabolically favorable effects. Using large-scale nationwide cohort data, we investigated our hypotheses.

## 2. Materials and Methods

### 2.1. Data Sources and Study Population

We conducted the study based on data extracted from databases of the National Health Insurance System (NHIS) of the National Health Insurance Corporation of South Korea [12]. NHIS covers approximately 97% of the South Korean population, while medical aid covers the remaining 3%. Eligibility data (age, sex, and socioeconomic variables), medical care utilization (diagnosis based on International Classification of Diseases 10th revision [ICD-10] codes, medical treatment, and medical expenses), and health examination results (results of a standardized medical examination provided biennially and questionnaires on lifestyle habits) were retrieved from the NHIS databases after approval of the study protocol by an official NHIS review committee.

**Figure 1:**
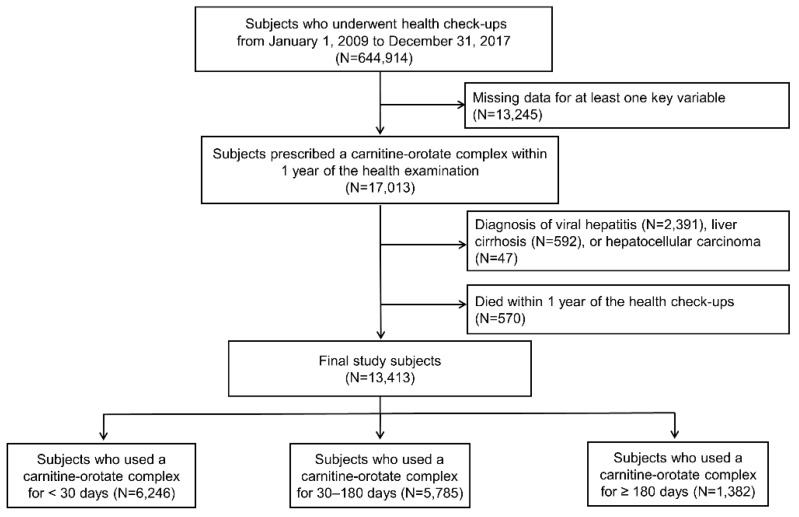
Flow chart of the study population.

Among 644,914 participants aged 20 years or older who underwent a health examination provided by the Korean National Health Insurance System between 2009 and 2017, 13,245 participants with missing data for key variables were excluded. We selected 17,013 participants who were prescribed carnitine-orotate complex within 1 year of their health examination. We then excluded 3030 participants with a preexisting diagnosis for the following diseases: LC as defined by a disease code of K703 or K746, viral hepatitis as defined by a disease code of B15–B19, or hepatocellular carcinoma (HCC) as defined by a disease code of C22.0. We further excluded 570 individuals who died within 1 year of enrollment. Finally, a total of 13,413 individuals were included in the analysis (Figure 1).

### 2.2. Definition and Classification of Carnitine-Orotate Complex Intake

Carnitine-orotate complex (Godex^®^, 412 mg/capsule; Celltrion Pharm, Seoul, Korea) is prescribed in South Korea for one of the following conditions: (1) serum level of aspartate transaminase (AST) or alanine transferase (ALT) ≥ 60 U/L, (2) serum level of AST or ALT ≥ 40 U/L for more than three months in cases of serum levels of AST or ALT of 40–60 U/L, (3) continuous administration depending on the patient’s condition or physician’s opinion even if AST or ALT are <40 U/L, or (4) patients with LC or HCC. Participants were classified according to the duration of use of carnitine-orotate complex: <30 days, 30–180 days, and ≥180 days. Participants who used carnitine-orotate complex within 1-year prior to enrollment were also identified.

### 2.3. Study Outcomes

All-cause mortality and incidence of LC and HCC were the primary outcomes. All-cause mortality was assessed by extracting data from the Korean National Statistical Office. Newly diagnosed LC was defined as a recording of ICD-10 codes K703 or K746, while HCC was defined as a recording of the ICD-10 code of C22.0. Composite study outcome was any of all-cause mortality, LC, or HCC. To further examine cause-specific mortality, cardiovascular mortality was defined as ICD-10 codes I00-78, respiratory mortality as ICD-10 codes J00-99, HCC mortality as ICD-10 code C22, and lung cancer mortality as ICD-10 code C34 based on the most common contributing causes of death among Koreans and the diseases of interest in this study. Study subjects were followed-up from the date of health examination as an index date to the date of LC, HCC, or death, or until December 31, 2019, whichever occurred first. The median follow-up duration for the study endpoint was 3.67 years (interquartile range: 2–6.25 years).

### 2.4. Other Measurements and Definitions

Body mass index (BMI) was calculated as body weight (kg) divided by height squared (m^2^). Obesity was defined as BMI ≥ 25 kg/m^2^ using World Health Organization recommendations for Asian populations [13]. Measurements of plasma glucose, triglycerides, total cholesterol, high-density lipoprotein cholesterol (HDL-C), low-density lipoprotein cholesterol triglycerides, AST, and ALT were performed after an overnight fast. Information on smoking status, alcohol consumption, physical activity, and health conditions was obtained using a self-report questionnaire. Subjects were categorized into non-smokers (ex-smokers and never smokers) and current smokers. Alcohol consumption status was categorized as <30 g of alcohol/day or ≥30 g/day [14,15], while regular physical activity was defined as performing moderate exercise for over 30 min per session more than five times per week or vigorous physical activity for over 20 min per session more than three times per week. Household income level was dichotomized at the lower 25%.

Baseline medical conditions were confirmed based on a combination of the results of the self-reported questionnaire, anthropometric and laboratory examinations, and prescription claims under related ICD-10 codes. Hypertension was defined as having at least one prescription claim for antihypertensive medications per year under ICD-10 codes I10–I13 and I15 or systolic/diastolic blood pressure (BP) ≥ 140/90 mmHg. Type 2 diabetes was defined as having at least one claim per year for the prescription of anti-diabetic medications under ICD-10 codes E11-14 or fasting plasma glucose level ≥ 126 mg/dL. Dyslipidemia was defined as having at least one prescription claim of lipid-lowering medications per year under ICD-10 code E78 or a total cholesterol level ≥ 240 mg/dL. Metabolic syndrome was diagnosed if at least three of the following conditions were met according to the modified criteria of the National Cholesterol Education Program Adult Treatment Panel III [16]: (1) waist circumference ≥90 cm for men or ≥85 cm for women [17]; (2) serum triglyceride level ≥ 150 mg/dL or use of lipid-lowering medications; (3) HDL-C level < 40 mg/dL for men or <50 mg/dL for women, or use of lipid-lowering medications; (4) systolic BP ≥ 130 mmHg, diastolic BP ≥ 85 mmHg, or use of an antihypertensive drug; and (5) FPG level ≥100 mg/dL or use of anti-diabetic medications. The presence of fatty liver disease was estimated by calculating the fatty liver index (FLI) using BMI, waist circumference, and levels of gamma-glutamyl transferase and triglycerides, with a cut-off value of 60 for hepatic steatosis and 30 for excluding it [18]. Finally, we used the Charlson comorbidity index (CCI) to identify different comorbid medical conditions, which were defined based on 19 chronic diseases using ICD-10 codes in the claims database [19].

### 2.5. Statistical Analyses

Participant characteristics were compared according to the duration of use of carnitine-orotate complex using analysis of variance for continuous variables and the χ^2^ test for categorical variables. According to the three categories of duration of use, multivariable Cox proportional hazards regression analysis was used to evaluate the association between the use of carnitine-orotate complex and the risk of the composite outcome, all-cause mortality, LC, and HCC, respectively; hazard ratios and 95% confidence intervals are reported. The proportional hazards assumption was verified using Schoenfeld residuals’ test. Model 1 was unadjusted; model 2 was adjusted for age and sex; model 3 was adjusted for age, sex, smoking status, alcohol consumption, physical activity, income, obesity, and Charlson comorbidity index; and model 4 was further adjusted for serum AST, ALT, and the use of carnitine-orotate complex within 1-year prior to enrollment. Competing events were treated as censored observations. The association between the use of carnitine-orotate complex and cause-specific mortality was also explored using a multivariable cox proportional hazards regression analysis. We performed subgroup analyses by age, sex, presence of obesity, metabolic syndrome, dyslipidemia, type 2 diabetes, fatty liver disease, and CCI to investigate the associations between the use of carnitine-orotate complex and the risk of mortality according to the duration of its use in these subgroups. *p*-values for interactions were calculated using Cox regression analyses. Statistical analyses were performed using SAS version 9.4 (SAS Institute Inc., Cary, NC, USA). Two-sided *p*-values < 0.05 were considered statistically significant.

## 3. Results

### 3.1. Baseline Characteristics of the Study Subjects

Baseline characteristics of the study population according to the duration of use of carnitine-orotate complex are shown in Table 1. Of the 13,413 subjects, 46.6% had used carnitine-orotate complex for <30 days, 43.1% for 30–180 days, and 10.3% for ≥180 days. Participants who had used carnitine-orotate complex for ≥180 days had the oldest mean age and were predominantly males and heavy drinkers (all *p*-values < 0.001). These subjects were more likely to be obese and have abdominal obesity, metabolic syndrome, type 2 diabetes, hypertension, dyslipidemia, fatty liver disease, a high CCI, and high systolic BP, diastolic BP, and serum levels of glucose and triglycerides than those subjects who used carnitine-orotate complex for a shorter duration (all *p*-values < 0.001). In addition, the proportion of individuals who had used carnitine-orotate complex within 1 year of enrollment was the highest in this group at 44.8% (*p*-value < 0.001).

### 3.2. The Risk of Mortality, LC, HCC, and the Composite Outcome According to the Duration of Carnitine-Orotate Complex Use

Table 2 presents the risks of all-cause mortality, LC, HCC, and the composite outcome according to the duration of use of carnitine-orotate complex. During a median follow-up of 3.67 years, 708 deaths, 372 LC cases, and 74 HCC cases were documented. After multivariable adjustment for age, sex, income, smoking status, alcohol consumption, physical activity, obesity, hypertension, dyslipidemia, and CCI, the multivariable-adjusted HR (95% CI) for all-cause mortality was 0.64 (95% CI 0.49–0.84) in participants who had used carnitine-orotate complex for ≥180 days and 0.63 (95% CI 0.52–0.74) for participants who had used carnitine-orotate complex for 30–180 days compared to those individuals who had used it for <30 days (*p* trend <0.001). Results were similar after further adjustment for serum AST, ALT, and the use of carnitine-orotate complex within 1-year prior to enrollment (*p* for trend <0.001). Similar to all-cause mortality, participants who had used carnitine-orotate complex for ≥180 days had a lower risk of cardiovascular death (0.29, 95% CI 0.11–0.72) than those who had used it for <30 days (*p* trend 0.01), but this was not significant for mortality from lung cancer or respiratory diseases (Table A1).

After adjusting for all variables, the adjusted HRs of LC and HCC incidences increased to 1.61 (95% CI 1.17–2.21) and 2.13 (95% CI 1.11–4.08), respectively, in participants who had used carnitine-orotate complex for ≥180 days compared to those who had used it for <30 days (*p* for trend 0.015, respectively). The null association was found for HCC mortality such that the adjusted HRs were 1.27 (95% CI 0.48–3.38) in individuals who had used carnitine-orotate complex for ≥180 days and 0.95 (95% CI 0.50–1.8) for those who had used it for 30–180 days compared to those who had used it for <30 days (*p* for trend 0.838) (Table A1). The risk of the composite outcome of all-cause mortality, LC, and HCC showed a significantly decreasing trend according to duration of carnitine-orotate complex use, similar to the trend observed for all-cause mortality (Table 2) (*p* for trend 0.001).

### 3.3. Subgroup Analysis

**Figure 2:**
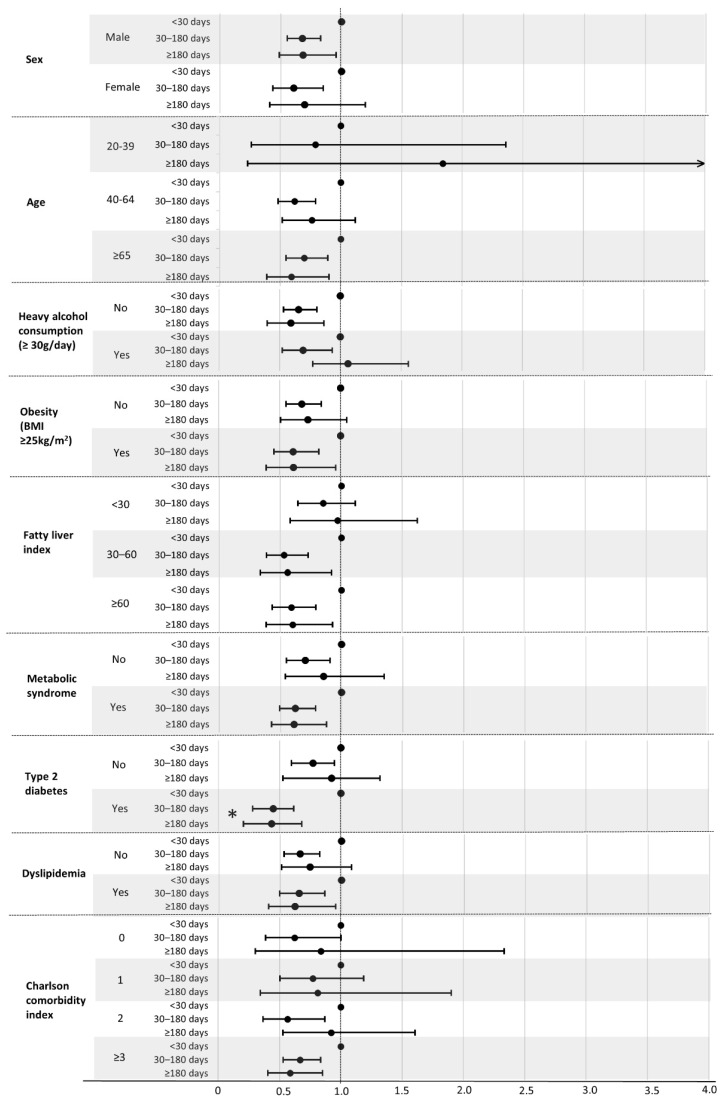
Subgroup analysis of all-cause mortality according to duration of carnitine-orotate complex use. HRs (95% CIs) were calculated using a multivariable Cox proportional hazard regression analysis after adjusting for age, sex, income, smoking status, alcohol consumption, physical activity, obesity, hypertension, dyslipidemia, Charlson comorbidity index, serum aspartate transaminase level, serum alanine transferase level, and use of carnitine-orotate complex within 1-year prior to enrollment.

Figure 2 shows the results of subgroup analysis for all-cause mortality according to the duration of use of carnitine-orotate complex. A decrease in mortality with the use of carnitine-orotate complex for ≥180 days was evident in older adults (adjusted HR of 0.59, 95% CI 0.39–0.90), males (adjusted HR 0.68, 95% CI 0.49–0.96), and non-to mild drinkers (adjusted HR 0.59, 95% CI 0.42–0.84). Likewise, the presence of obesity, fatty liver, metabolic syndrome, type 2 diabetes, dyslipidemia, and CCI ≥ 3 was associated with a reduced risk of all-cause mortality after adjustment for all potential confounders (adjusted HRs [95% CI] of 0.61 [0.39–0.96] for individuals with obesity; 0.60 [0.39–0.93] for those with fatty liver; 0.61 [0.43–0.88] for those with metabolic syndrome; 0.43 [0.28–0.68] for those with type 2 diabetes; 0.62 [0.41–0.95] for those with dyslipidemia; and 0.59 [0.40–0.85] for those with CCI ≥ 3). Among these comorbidities, a significant interaction was observed only in the type 2 diabetes subgroup (*p* for interaction 0.001). By contrast, mortality according to longer use of carnitine-orotate complex was not significantly lower than that of shorter use in females, individuals under 65 years of age, heavy drinkers, non-obese adults, and individuals without obesity, fatty liver, metabolic syndrome, type 2 diabetes, or dyslipidemia, or those with a low number of comorbidities.

## 4. Discussion

In this nationwide study, prolonged use of carnitine-orotate complex was associated with a lower all-cause mortality rate of more than 30% after adjustment for potential confounders compared to shorter use. In addition, a significant inverse relationship was found in cardiovascular mortality, albeit with a small number of events. The composite outcome risk of all-cause mortality, LC, and HCC incidence showed a decreasing trend with longer use of carnitine-orotate complex but the risks of LC and HCC incidence showed increasing trends. No significant association was found in the case of HCC mortality. Notably, the risk of mortality according to long-term use of carnitine-orotate complex was significantly lower in individuals with metabolic risk factors such as obesity, fatty liver, metabolic syndrome, dyslipidemia, and type 2 diabetes, with the greatest risk reduction (>50%) observed among individuals with type 2 diabetes. Interestingly, the beneficial effect of prolonged use of carnitine-orotate complex on mortality was attenuated in heavy drinkers.

The mechanism by which carnitine-orotate complex affects the risk of mortality can be explained in part by the role of L-carnitine. L-carnitine is a key player in mitochondrial fatty acid β-oxidation [20]. Insufficient levels of carnitine suppress mitochondrial β-oxidation, and oxidative stress occurs subsequently due to an increase in reactive oxygen species (ROS) leading to mitochondrial damage and a higher apoptosis rate [21,22,23]. Molecular mechanisms linking oxidative stress and the resulting deleterious health effects such as cancer, stroke, and others have been established [24,25]. One study reported that use of carnitine-orotate complex reduced overall oxidative stress and increased mitochondrial DNA copy number [4,26]. Use of carnitine-orotate complex may reduce intracellular ROS levels and thus delay immune cell death. In addition, other components of carnitine-orotate complex likely contribute to its beneficial effects on mortality, i.e., biphenyl dimethyl dicarboxylate is a metabolic activator [27,28] and adenine and adenosine enhance β-oxidation [29,30,31], resulting in strong antioxidant activity and a protective effect against cell damage [4].

Several earlier studies have reported that L-carnitine has mortality benefits [8,9,10,11]. One systematic review and meta-analysis suggested that L-carnitine supplementation was associated with a 27% reduction in all-cause mortality in patients who had experienced an acute myocardial infarction [8]. Another systemic review suggested that at least 2 g/day of carnitine supplementation may help to reduce mortality risk in patients with septic shock [9]. Low-dose L-carnitine treatment of 500 mg/day for more than six months also resulted in improved cardiac morbidity and mortality in patients on hemodialysis [10]. Of note, we confirmed the beneficial effects of long-term use of carnitine-orotate complex at about 450 mg/day as L-carnitine. There may exist differences between previous studies and our study, such as the existence of certain stressors that can cause secondary carnitine deficiency and achievement of supra-physiologic plasma level of L-carnitine or not. Additionally, information about its ability to correct carnitine deficiency as carnitine orotate complex, the most absorbable form of L-carnitine, is yet insufficient. Additional experimental studies and randomized controlled studies should confirm our findings on the role of long-term exposure to carnitine-orotate complex on the improved mortality.

Meanwhile, we observed an increased risk of developing LC and HCC in those individuals who had used carnitine-orotate complex for longer periods. This may be the case because those individuals had been more likely to have advanced chronic liver diseases or strong clinical requirements for continuous supplementation, i.e., they already had other baseline risk factors for developing LC and HCC. We confirmed that mortality from HCC was not significantly increased in this group, although this finding should be interpreted with caution because of the small number of cases. On the other hand, there are some evidence on orotate that it induced endothelial dysfunction, hepatic steatosis, or insulin resistance in vitro models [32,33]. It is possible that the orotate component of the carnitine-orotate complex counteracted the beneficial effects of carnitine, which could affect the study outcome, such as an increased risk of LC or HCC. However, in a recent study, patients with LC who received L-carnitine supplementation for one month or longer had a 34% lower mortality rate than those who did not [11]. It is assumed that long-term use of carnitine-orotate complex may reduce mortality in those who currently have LC or are at high risk of developing LC or HCC. However, further studies with larger sample sizes and longer treatment durations are needed to test this hypothesis.

Interestingly, the effects of long-term carnitine-orotate complex use on mortality were more pronounced in individuals with metabolic syndrome, dyslipidemia, fatty liver, obesity, and type 2 diabetes. These individuals with metabolic risk factors are in a state of insulin resistance as a shared pathophysiology [34,35]. Oxidative stress and mitochondrial dysfunction are crucial for the disease process in people with fatty liver disease [36,37] as well as for immunological dysfunction in patients with type 2 diabetes [38]. According to several clinical trials, patients with type 2 diabetes have lower plasma levels of free carnitine than healthy individuals [39,40]. Above all, the metabolic beneficial actions of carnitine has been shown to affect fatty acid oxidation rate and tissue insulin sensitivity [5,41,42]. Long-term intake of mitochondria-targeting nutrients may mitigate the risk of mortality by alleviating the impairment of insulin signaling cascade and its pathological consequences as well as modulating oxidative stress and altered immune function in individuals with metabolic risk factors.

Analysis stratified by age revealed that the improvement in mortality associated with long-term use of carnitine-orotate complex was noticeable in elderly. As carnitine is naturally present only in meat and dairy products [43], except for synthesized in the liver and kidneys, older adults are at risk for carnitine deficiency due to insufficient intake. A longer period of carnitine supplementation may therefore provide benefit to elderly individuals who are at risk of carnitine deficiency compared to younger individuals. Improvement in mortality was also more evident in non-to mild drinkers than in heavy drinkers. This may be due to the combination of decreased biosynthesis of carnitine in the liver and damage to multiple organs and systems from prolonged ethanol drinking in addition to decreased dietary intake by heavy drinkers [44].

Our results should be interpreted with caution. Due to the retrospective cohort design of our study, reverse causality might exist between the use of carnitine-orotate complex and the risk of mortality, although we included a 1-year lag period in our study. Operational diagnosis using claim database may result in a possibility of misdiagnosis. Data regarding nutritional status or information concerning other supplements were also unavailable. Despite extensive consideration of potential confounders, residual confounders may still have been present in our dataset. Furthermore, the study population was a homogenous population of Koreans; thus, the generalizability of our findings to other populations or ethnic groups is unclear. Additionally, due to the small number of events, it was difficult to prespecify cause-specific mortality. Thus, additional caution is needed to generalize the results of cause-specific mortality even though we found a significant inverse association for cardiovascular mortality.

Despite these limitations, this is the first study to report improved mortality from longer use of carnitine-orotate complex based on analyses of nationwide cohort data. Of note, we observed stronger risk reduction in individuals with metabolic risk factors such as the presence of obesity, fatty liver, dyslipidemia, type 2 diabetes, and metabolic syndrome with prolonged use of carnitine-orotate complex while the effect was weaker in heavy drinkers than in non- to mild-drinkers. Based on our findings, we recommend continuous supplementation with carnitine-orotate complex, especially in individuals at high metabolic risk, and recommend abstinence from alcohol in individuals taking carnitine-orotate complex. Further randomized controlled trials with larger cohorts are needed to confirm the survival benefits of carnitine-orotate complex supplementation.

## 5. Conclusions

In this population-based cohort study based on analyses of health examination data and history of use of carnitine-orotate complex, we found that a longer period of carnitine-orotate complex use was associated with a lower mortality rate compared to a shorter period of use. Importantly, the risk reduction was prominent in participants with metabolic risk factors such as obesity, fatty liver, dyslipidemia, metabolic syndrome, and type 2 diabetes.

## Figures and Tables

**Table 1:** Baseline characteristics of participants using carnitine-orotate complex according to duration of use.

	Duration of Carnitine-Orotate Complex Use				*p*-Value
	Total	<30 Days	30–180 Days	≥180 Days	
*n*	13,413	6246	5785	1382	
Age, years	51.97 ± 12.94	52.27 ± 13.79	51 ± 12.24	54.63 ± 11.26	<0.001
Age, *n* (%)					<0.001
20–39	2390 (17.8)	1210 (19.4)	1059 (18.3)	121 (8.8)	
40–64	8770 (65.4)	3833 (61.4)	3932 (68.0)	1005 (72.7)	
≥65	2253 (16.8)	1203 (19.2)	794 (13.7)	256 (18.5)	
Male sex, *n* (%)	8769 (65.4)	3794 (60.7)	3985 (68.9)	990 (71.6)	<0.001
Current smoker, *n* (%)	3833 (28.6)	1710 (27.4)	1720 (29.7)	403 (29.2)	<0.001
Heavy alcohol drinker, *n* (%)	1844 (13.8)	715 (11.5)	888 (15.4)	241 (17.4)	<0.001
Regular exerciser, *n* (%)	2542 (19.0)	1190 (19.1)	1103 (19.1)	249 (18.0)	0.645
Low income, *n* (%)	2064 (15.4)	976 (15.6)	859 (14.9)	229 (16.6)	0.218
BMI, kg/m^2^	25.6 ± 3.9	24.9 ± 3.7	25.99 ± 3.83	26.6 ± 4.04	<0.001
Waist circumference, cm	85.85 ± 9.79	84.07 ± 9.57	87.02 ± 9.66	89.05 ± 9.77	<0.001
Systolic BP, mmHg	126.64 ± 15.2	125.42 ± 15.32	127.2 ± 14.9	129.77 ± 15.32	<0.001
Diastolic BP, mmHg	78.99 ± 10.39	78.09 ± 10.34	79.59 ± 10.37	80.56 ± 10.33	<0.001
Total cholesterol, mg/dL	200.2 ± 42.93	199.67 ± 40.91	202.97 ± 43.94	190.94 ± 46.11	<0.001
Triglycerides, mg/dL ^a^	141 (95–210)	127 (86–189)	152 (104–223)	156 (109–232)	<0.001
HDL cholesterol, mg/dL	52.88 ± 17.58	54.0 ± 17.8	51.9 ± 18	51.4 ± 14.3	<0.001
LDL cholesterol, mg/dL	114.77 ± 40.25	115.7 ± 37.5	116.4 ± 42.3	103.7 ± 41.5	<0.001
Fasting glucose, mg/dL	108.2 ± 32.44	104.1 ± 28.9	110 ± 33.5	119.4 ± 38.8	<0.001
Aspartate transaminase, U/L ^a^	35 (25–54)	31 (19–58)	53 (30–82)	51 (28–80)	<0.001
Alanine transferase, U/L ^a^	42 (23–72)	29 (22–43)	40 (28–60)	47 (33–68)	<0.001
Metabolic syndrome	7837 (58.4)	3072 (49.2)	3674 (63.5)	1091 (78.9)	<0.001
Hypertension	6125 (45.7)	2480 (39.7)	2722 (47.1)	923 (66.8)	<0.001
Diabetes mellitus	3042 (22.7)	1028 (16.5)	1408 (24.3)	606 (43.9)	<0.001
Dyslipidemia	5733 (42.7)	2194 (35.1)	2698 (46.6)	841 (60.9)	<0.001
Fatty liver index					<0.001
0–30	4009 (29.9)	2580 (41.31)	1239 (21.42)	190 (13.75)	
30–60	3863 (28.8)	1763 (28.23)	1722 (29.77)	378 (27.35)	
≥60	5541 (41.3)	1903 (30.47)	2824 (48.82)	814 (58.9)	
Charlson comorbidity index					<0.001
0	2796 (20.9)	1592 (25.5)	1092 (18.9)	112 (8.1)	
1	3306 (24.7)	1527 (24.5)	1531 (26.5)	248 (18.0)	
2	2738 (20.4)	1151 (18.4)	1245 (21.5)	342 (24.8)	
≥3	4573 (34.1)	1976 (31.6)	1917 (33.1)	680 (49.1)	
Use of carnitine-orotate complex within 1-year prior to the health check-up, *n* (%)	1879 (14.0)	346 (5.5)	914 (15.8)	619 (44.8)	

Data are presented as means ± standard deviations or numbers (percentages). Abbreviation: BMI, body mass index; BP, blood pressure; HDL, high-density lipoprotein; LDL, low-density lipoprotein ^a^ Presented as medians (interquartile ranges) using the Wilcoxon rank-sum test.

**Table 2:** HRs (95% CIs) of the risks of all-cause mortality, LC, HCC, and the composite outcome according to the duration of use of carnitine-orotate complex.

	Total (*n*)	Events (*n*)	Person Years	IR ^a^	HR (95% CI)			
					Model 1 ^b^	Model 2 ^c^	Model 3 ^d^	Model 4 ^e^
All-cause mortality								
<30 days	6246	441	27,299	16.15	1 (reference)	1 (reference)	1 (reference)	1 (reference)
30–180 days	5785	203	24,317	8.35	0.51 (0.44–0.61)	0.59 (0.50–0.70)	0.63 (0.52–0.74)	0.66 (0.55–0.79)
≥180 days	1382	64	5501	11.63	0.72 (0.55–0.93)	0.63 (0.48–0.82)	0.64 (0.49–0.84)	0.69 (0.51–0.92)
*p* for trend					<0.001	<0.001	<0.001	<0.001
LC								
<30 days	6246	137	26,862	5.10	1 (reference)	1 (reference)	1 (reference)	1 (reference)
30–180 days	5785	163	23,855	6.83	1.34 (1.06–1.68)	1.37 (1.09–1.72)	1.44 (1.14–1.81)	1.14 (0.90–1.44)
≥180 days	1382	72	5310	13.56	2.64 (1.98–3.51)	2.35 (1.77–3.13)	2.49 (1.84–3.37)	1.61 (1.17–2.21)
*p* for trend					<0.001	<0.001	<0.001	0.015
HCC								
<30 days	6246	28	27,237	1.03	1 (reference)	1 (reference)	1 (reference)	1 (reference)
30–180 days	5785	26	24,256	1.07	1.05 (0.62–1.79)	1.08 (0.63–1.84)	1.12 (0.65–1.92)	0.85 (0.49–1.47)
≥180 days	1382	20	5462	3.66	3.61 (2.04–6.41)	2.94 (1.65–5.22)	2.99 (1.63–5.49)	2.13 (1.11–4.08)
*p* for trend					<0.001	<0.001	<0.001	0.015
Composite all-cause mortality, LC, and HCC								
<30 days	6246	559	26,828	20.84	1 (reference)	1 (reference)	1 (reference)	1 (reference)
30–180 days	5785	348	23,827	14.61	0.70 (0.61–0.80)	0.77 (0.67–0.88)	0.81 (0.71–0.93)	0.76 (0.66–0.87)
≥180 days	1382	123	5293	23.24	1.10 (0.91–1.34)	0.97 (0.80–1.18)	0.99 (0.82–1.22)	0.84 (0.67–1.04)
*p* for trend					<0.001	0.001	0.008	0.001

HRs (95% CIs) were calculated using multivariable Cox proportional hazard regression analysis. ^a^ Incidence per 1000 person years. ^b^ Model 1 was unadjusted. ^c^ Model 2 was adjusted for age and sex. ^d^ Model 3 was adjusted for age, sex, income, smoking status, alcohol consumption, physical activity, obesity, obesity, hypertension, dyslipidemia, and Charlson comorbidity index. ^e^ Model 4 was adjusted for age, sex, income, smoking status, alcohol consumption, physical activity, obesity, obesity, hypertension, dyslipidemia, Charlson comorbidity index, serum aspartate transaminase, serum alanine transferase, and the use of carnitine-orotate complex within 1-year prior to enrollment. Abbreviation: HR, hazard ratio; CI, confidence interval; IR, incidence rate; LC, liver cirrhosis; HCC, hepatocellular carcinoma.

## Data Availability

This study was performed using the National Health Insurance System database, and the results do not necessarily represent the opinion of the National Health Insurance Corporation. Restrictions apply to the availability of these data, which were used under license for this study.

## Appendix A

**Table A1:** HRs (95% CIs) of the risks of cause-specific mortality according to duration of use of carnitine-orotate complex.

	Total (*n*)	Events (*n*)	Person Years	IR ^a^	HR (95% CI)		
					Model 1 ^b^	Model 2 ^c^	Model 3 ^d^
Cardiovascular mortality							
<30 days	6246	65	27,311	2.38	1 (reference)	1 (reference)	1 (reference)
30–180 days	5785	30	24,390	1.23	0.64 (0.42–1.00)	0.64 (0.39–0.97)	0.61 (0.39–0.97)
≥180 days	1382	6	5505	1.09	0.42 (0.18–0.96)	0.38 (0.16–0.88)	0.29 (0.11–0.72)
*p* for trend					0.032	0.023	0.010
Respiratory mortality							
<30 days	6246	38	27,338	1.39	1 (reference)	1 (reference)	1 (reference)
30–180 days	5785	13	24,528	0.53	0.51 (0.27–0.97)	0.52 (0.27–0.99)	0.54 (0.28–1.06)
≥180 days	1382	5	5495	0.91	0.60 (0.23–1.52)	0.55 (0.21–1.43)	0.53 (0.18–1.56)
*p* for trend					0.091	0.094	0.157
HCC mortality							
<30 days	6246	22	27,160	0.81	1 (reference)	1 (reference)	1 (reference)
30–180 days	5785	19	24,359	0.78	1.08 (0.58–2.00)	1.11 (0.59–2.09)	0.95 (0.50–1.8)
≥180 days	1382	6	5505	1.09	1.17 (0.48–2.89)	1.15 (0.45–2.92)	1.27 (0.48–3.38)
*p* for trend					0.937	0.929	0.838
Lung cancer mortality							
<30 days	6246	39	27,273	1.43	1 (reference)	1 (reference)	1 (reference)
30–180 days	5785	18	24,324	0.74	0.58 (0.33–1.02)	0.65 (0.37–1.15)	0.79 (0.44–1.43)
≥180 days	1382	5	5495	0.91	0.53 (0.21–1.34)	0.61 (0.23–1.58)	0.82 (0.29–2.35)
*p* for trend					0.102	0.257	0.732

HRs (95% CIs) were calculated using multivariable Cox proportional hazard regression analysis. ^a^ Incidence per 1000 person years. ^b^ Model 1 was adjusted for age and sex. ^c^ Model 2 was adjusted for age, sex, income, smoking status, alcohol consumption, physical activity, obesity, obesity, hypertension, dyslipidemia, and Charlson comorbidity index. ^d^ Model 3 was adjusted for age, sex, income, smoking status, alcohol consumption, physical activity, obesity, obesity, hypertension, dyslipidemia, Charlson comorbidity index, serum aspartate transaminase, serum alanine transferase, and the use of carnitine-orotate complex within 1-year prior to enrollment. Abbreviation: HR, hazard ratio; CI, confidence interval; IR, incidence rate; HCC, hepatocellular carcinoma.
